# A comparison of the epidemiology and outcomes of true refractory and recurrent ventricular fibrillation out-of-hospital cardiac arrest: a retrospective study

**DOI:** 10.1016/j.resplu.2026.101302

**Published:** 2026-03-27

**Authors:** Abdulrahman Alhenaki, Zainab Alqudah, Timothy Kennett, Ashanti Dantanarayana, Brett Williams, Ziad Nehme

**Affiliations:** a Department of Paramedicine, Monash University, Frankston, Victoria, Australia; b Prince Sultan ibn Abdulaziz for Emergency Medical Services, King Saud University, Riyadh, Saudi Arabia; c Faculty of Allied Medical Sciences, Jordan University of Science and Technology, Irbid, Jordan; d Imam Abdulrahman Bin Faisal University, Dammam, Saudi Arabia; e Centre for Research and Evaluation, Ambulance Victoria, Blackburn North, Victoria, Australia; f School of Public Health and Preventive Medicine, Monash University, St Kilda, Victoria, Australia

**Keywords:** Defibrillation, Emergency medical services, Out-of-hospital cardiac arrest, Resuscitation, Ventricular fibrillation

## Abstract

**Aim:**

To explore and compare patient characteristics and clinical outcomes across true-refractory and recurrent VF/pVT cases, at two commonly used time points, including after the first and third consecutive shocks.

**Methods:**

We performed a retrospective analysis of adult (≥16 years old) out-of-hospital cardiac arrest (OHCA) patients with initially shockable rhythms and complete defibrillator recordings between January 2019 and October 2022. Defibrillator recordings were reviewed using standardised criteria to classify patients into true-refractory VF/pVT, defined as persistent VF/pVT 5 s after shock delivery, and recurrent VF/pVT, defined as termination of arrhythmia at 5 s post-shock followed by recurrence at the end of the 2-minute CPR cycle. Multivariable logistic regression was used to examine the impact of true-refractory VF/pVT on survival outcomes and to identify factors associated with true-refractory VF/pVT.

**Results:**

Of the 2343 cases with complete defibrillator recordings, 1880 cases met the inclusion criteria. The incidence of true-refractory VF/pVT declined with increasing defibrillation attempts, accounting for 28.9%, 5.0%, and 1.0% of cases after the first, third and fifth consecutive shocks, respectively. Compared to patients with recurrent VF/pVT, true-refractory VF/pVT was associated with lower odds of survival to hospital discharge (adjusted odds ratio (AOR) 0.45, 95% CI: 0.22–0.92; *p* = 0.029) and pre-hospital ROSC (AOR 0.58, 95% CI: 0.36–0.95; *p* = 0.029). Older age and prolonged resuscitation were associated to true-refractory cases.

**Conclusion:**

Although associated with substantially worse outcomes, true-refractory VF/pVT is a time-limited condition, which typically responds to repeated defibrillation attempts. Further research is needed to determine whether earlier recognition and optimal management of true-refractory VF/pVT can improve outcomes.

## Introduction

Approximately one-quarter of out-of-hospital cardiac arrests (OHCA) present with initial shockable rhythms of Ventricular Fibrillation (VF) or pulseless Ventricular Tachycardia (pVT).^1^ In these cases, better survival outcomes have been reported.^1^ However, nearly half of all initial VF/pVT cases progress to refractory VF/pVT**,** where arrhythmia persists despite multiple defibrillation attempts.^2^, ^3^

The reported incidence of refractory VF/pVT in OHCA varies widely, largely reflecting differences in the definitions applied across observational studies and major clinical trials.^3^, ^4^, ^5^, ^6^, ^7^, ^8^ The ALPS trial defined refractory VF/pVT as ventricular arrhythmia persisting after at least one defibrillation attempt.^5^ In contrast, the ARREST and DOSE-VF trials defined refractory VF/pVT as an arrhythmia persisting after three consecutive shocks.^7^, ^8^ These inconsistencies in defining refractory VF/pVT limit the development and testing of targeted and novel interventions for this population. Furthermore, both definitions ignore the distinction between true-refractory and recurrent VF/pVT. Differentiating between these VF phenotypes is clinically important, as each may influence shock success and carry distinct prognostic implications.^9^, ^10^, ^11^

Current resuscitation guidelines call for immediate CPR after defibrillation, with rhythm reassessment delayed until after a 2-minute cycle of CPR. This approach does not distinguish between recurrent VF/pVT, defined as termination of VF/pVT at least five seconds post-shock followed by its spontaneous recurrence, and true-refractory VF/pVT, where VF/pVT persists continuously before and after shock delivery for the first three shocks.^12^ Real-time differentiation is challenging because it requires reviewing electrocardiogram recordings to accurately verify the rhythm response to each shock.^13^ These groups likely represent distinct clinical patterns; however, no study has directly compared their baseline characteristics or outcomes, leaving this distinction poorly defined.

This study aims to explore and compare patient characteristics and clinical outcomes across recurrent and true-refractory VF/pVT cases, at two commonly used time points, including after the first and third consecutive shocks. In addition, the study examines factors associated with the development of true-refractory VF/pVT and its impact on survival outcome.

## Methods

### Study design

We performed a retrospective cohort study using data from the Victorian Ambulance Cardiac Arrest Registry (VACAR). We included all adult (≥16 years old) OHCA patients with initially shockable rhythms who received an attempted resuscitation by emergency medical services (EMS) between January 2019 and October 2022. We excluded cases involving trauma, those with missing defibrillator recordings, and cases initially defibrillated by bystanders or first responders (as defibrillator recordings were not captured in these cases). The Monash University Human Research Ethics Committee approved the study (Project ID: 36838).

### Setting

Victoria is a state in Australia with over 6.6 million residents living in an area of 227,500 square kilometres. Ambulance Victoria, the state-wide EMS system, responds to more than 7000 OHCA every year.^14^ Ambulance Victoria has three levels of response to a suspected OHCA, including first responders with Basic Life Support skills, Advanced Life Support paramedics who can perform laryngeal mask airway insertion and administer intravenous adrenaline, and intensive care paramedics capable of inserting endotracheal tubes and who have an expanded range of medication options. Resuscitation guidelines follow the recommendations of the Australian and New Zealand Committee on Resuscitation (https://www.anzcor.org). Paramedics are authorised to cease resuscitation after 45 min of advanced life support if the patient does not develop return of spontaneous circulation (ROSC), has a non-shockable rhythm, has no signs of life (i.e., no gasps or pupillary reaction), and has no evidence of hypothermia or drug overdose.^15^ During the study period, no specific protocols were targeting the treatment of refractory VF/pVT patients. Clinical practice guidelines recommended standard defibrillation for refractory VF/pVT and administration of amiodarone after the 3rd and 5th consecutive shocks, or for recurrent VF/pVT (intensive care paramedics only). Adrenaline is typically administered after the second defibrillation attempt and repeated every second CPR cycle (approximately every 3–5 min).

### Data source and definitions

Data for this study were obtained from the VACAR, which documents all OHCA incidents attended by EMS in Victoria. The methodology of the VACAR has been described in detail elsewhere.^14^ VACAR collects over 150 data elements, including the Utstein-style descriptors and patient discharge outcomes from more than 100 participating hospitals. CPR quality and defibrillation data were recorded from the compression acceleration signal and impedance measurements captured by a single defibrillator (X series defibrillators, Zoll Medical). In this study, a senior clinical reviewer reviewed all defibrillator recordings and applied standardised rhythm definitions for all shocks (supplementary Table S1). At every shock, we determined whether (a) the rhythm was shockable and (b) whether the shock delivered resulted in successful defibrillation, defined as the termination of VF/pVT at 5 s after shock delivery (i.e. presence of asystole or organised rhythm). Cases of true-refractory VF/pVT were those with persistence of VF/pVT within 5 s of the shock being delivered. Recurrent VF/pVT cases were defined as those with successful defibrillation (termination of VF/pVT at 5 s) but had a recurrence of the VF/pVT at the end of the two-minute cycle of CPR. While these definitions were applied after every shock, they are categorised in this study after either the first or third consecutive shock, consistent with earlier studies.^5^, ^6^, ^7^ Patients with three true-refractory shocks required evidence of persistent VF/pVT after all three shocks.

### Study outcomes

The primary outcome of this study was survival to hospital discharge. The secondary outcome was ROSC and event survival.

### Data analysis

Data were organised and analysed using STATA statistical software 18 (StataCorp, 2015, College Station, TX). Arrest characteristics and survival outcomes are presented as frequencies and proportions for categorical variables and median and interquartile range (IQR) for continuous variables. We compared the baseline characteristics of recurrent and true-refractory VF/pVT patients after the first and third consecutive shocks. Comparisons were performed using the *χ*^2^ test or the Mann-Whitney *U* test, as appropriate.

Multivariable logistic regression models were used to identify: (1) the impact of true-refractory VF/pVT compared to recurrent VF/pVT on the study outcomes; and (2) factors associated with true-refractory VF/pVT after the first and third consecutive defibrillations. Based on previous literature,^16^, ^17^ models were adjusted for: age, male sex, arrest aetiology, bystander CPR, witness status, public location, metropolitan region, EMS response time, and resuscitation duration.

## Results

### Sample population

**Fig. 1:**
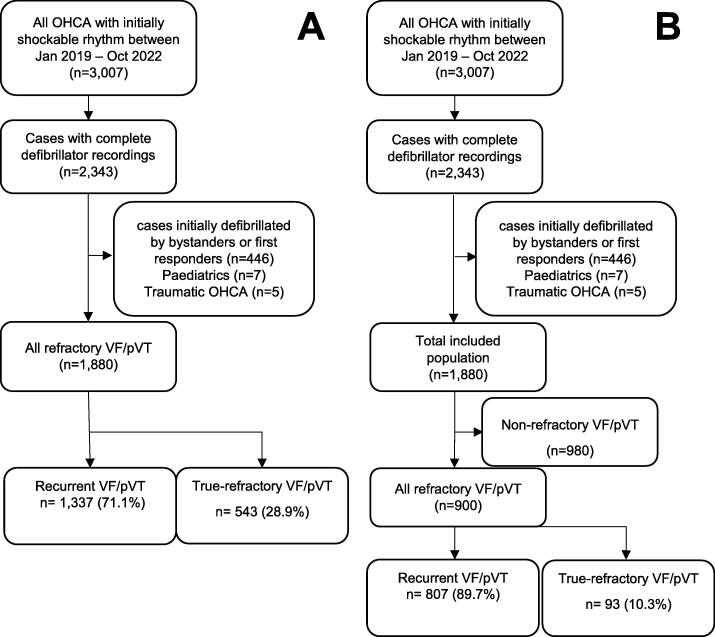
**Flow chart of included population. (A) After one shock. (B) After three shocks**.

Between January 2019 and October 2022, there were 3007 cases of initial VF/pVT receiving an EMS attempted resuscitation, of which 2343 (77.9%) had complete defibrillator recordings available. Of these, 1880 met the inclusion criteria for the study (Fig. 1).

### Frequency of true-refractory and recurrent VF/VT

After the first shock, 543 (28.9%) patients were defined as having true-refractory VF/pVT, while 1337 (71.1%) patients were classified as recurrent VF/pVT. Across subsequent shocks, the proportion of patients remaining in refractory VF/pVT declined substantially. A total of 900 (47.9%) patients received three consecutive shocks, of whom 807 (89.7%) met the criteria for recurrent VF/pVT, while only 93 (10.3%) were classified as true-refractory VF/pVT (i.e. defibrillation failure for three consecutive shocks).

**Fig. 2:**
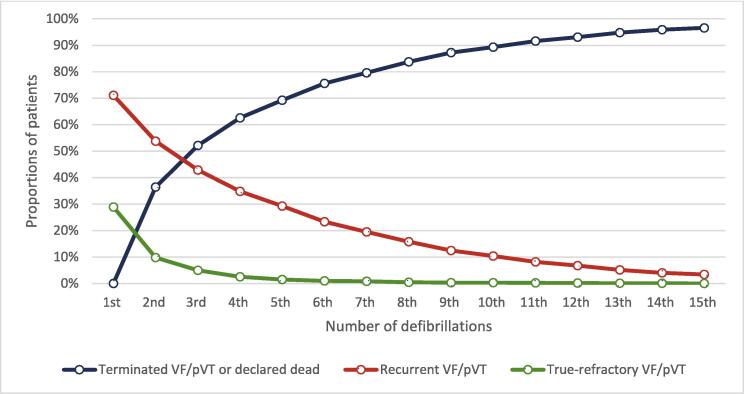
**Proportions of true-refractory and recurrent VF/pVT by number of defibrillations**.

Fig. 2 illustrates the proportion of true-refractory and recurrent VF/pVT across the number of defibrillations. After three consecutive defibrillations, 52.1% of cases had terminated VF/pVT or were declared dead, 42.9% of cases were classified as recurrent VF/pVT, whereas only 5.0% were true-refractory VF/pVT. After five shocks, the proportion of true-refractory VF/pVT declined to 1.0%, with no cases remaining after 15 consecutive shocks.

### Baseline characteristics

The baseline characteristics and outcomes of true-refractory and recurrent VF/pVT after one shock are described in Table 1. Compared to recurrent VF/pVT, patients with true-refractory VF/pVT were significantly more likely to be male (82.3% vs 77.9%, *p* = 0.031), were less often EMS-witnessed (10.3% vs 29.4%, *p* < 0.001), more frequently witnessed by bystanders (66.8% vs 54.3%, *p* < 0.001) and be more likely to arrest in private residences (70.2% vs 63.1%, *p* = 0.003). Resuscitation duration was considerably longer in the true-refractory compared to the recurrent VF/pVT group (median 33 min vs 15 min, *p* < 0.001). Regarding survival outcomes, patients with true-refractory VF/pVT had lower pre-hospital ROSC (57.1% vs 73.7%, *p* < 0.001), event survival (45.1% vs 67.9%, *p* < 0.001), and survival to hospital discharge (20.8% vs 47.8%, *p* < 0.001) compared to recurrent VF/pVT cases.

**Table 1:** Baseline characteristics and outcomes of true-refractory and recurrent VF/pVT patients after one shock.

**Variables**	**Cases with one shock** **(*n* = 1880)**	**True-refractory VF/pVT** **(*n* = 543)**	**Recurrent VF/pVT** **(*n* = 1337)**	***P*-value**
Age, median (IQR)	65 (54–75)	66 (55–75)	64 (53–75)	0.203
**Age, *n* (%)**				
16–30	43 (2.3)	7 (1.3)	36 (2.7)	0.065
31–45	173 (9.2)	40 (7.4)	133 (10.0)	0.079
46–60	553 (29.4)	163 (30.0)	390 (29.2)	0.714
61–75	649 (34.5)	203 (37.4)	446 (33.4)	0.096
>75	462 (24.6)	130 (23.9)	332 (24.8)	0.684
Male sex, *n* (%)	1488 (79.2)	447 (82.3)	1041 (77.9)	0.031
Presumed cardiac aetiology, *n* (%)	1811 (96.3)	518 (95.4)	1293 (96.7)	0.170
Bystander CPR, *n* (%)*	1199 (63.8)	394 (81.1)	799 (85.0)	0.057
**Witness status, *n* (%)**				
EMS	448 (23.9)	56 (10.3)	392 (29.4)	<0.001
Bystander	1085 (57.9)	362 (66.8)	723 (54.3)	<0.001
**Arrest location, *n* (%)**				
Private residence	1224 (65.1)	381 (70.2)	843 (63.1)	0.003
Public location	339 (18.0)	87 (16.0)	252 (18.9)	0.149
Aged care^a^	43 (2.3)	12 (2.2)	31 (2.3)	0.886
Other	274 (14.6)	63 (11.6)	211 (15.8)	0.020
Metropolitan region, *n* (%)	1241 (66.1)	354 (65.2)	887 (66.4)	0.604
**Time variables, median (IQR)**				
EMS response time	8.4 (6.5–11.7)	8.3 (6.4–11.1)	8.5 (6.5–12.0)	0.050
Resuscitation duration	21 (6–41)	33 (18–45)	15 (4–37)	<0.001
**Scene outcomes, *n* (%)**				
Died at scene or transit	640 (34.0)	262 (48.3)	378 (28.3)	<0.001
Transported with CPR	88 (4.7)	39 (7.2)	49 (3.7)	0.001
Transported with ROSC	1152 (61.3)	242 (44.6)	910 (68.1)	<0.001
**Survival outcomes, *n* (%)**				
Pre-hospital ROSC	1295 (68.9)	310 (57.1)	985 (73.7)	<0.001
Event survival	1153 (61.3)	245 (45.1)	908 (67.9)	<0.001
Discharged alive	741 (40.0)	112 (20.8)	629 (47.8)	<0.001

CPR, cardiopulmonary resuscitation; IQR, inter-quartile range; EMS, emergency medical services, ROSC, return of spontaneous circulation.

* Exclude EMS-witnessed cases.

a Refers to residential aged care facilities.

The baseline characteristics and outcomes of true-refractory and recurrent VF/pVT after three consecutive shocks are described in Table 2. Compared to recurrent VF/pVT, patients with true-refractory VF/pVT were more commonly aged >75 years (21.8% vs 31.2%, *p* = 0.041), less often arrested in public locations (10.8% vs 20.3%, *p* = 0.027) and had longer resuscitation duration (median 36 min vs 32 min, *p* = 0.037). Survival outcomes were all significantly lower for true-refractory VF/pVT cases compared to recurrent VF/pVT, including pre-hospital ROSC (48.4% vs 64.3%, *p* = 0.003), event survival (39.8% vs 52.8%, *p* = 0.018), and survival to hospital discharge (14.0% vs 32.2%, *p* < 0.001).

**Table 2:** Baseline characteristics and outcomes of true-refractory and recurrent VF/pVT patients after three consecutive shocks.

**Variables**	**Cases with 3 consecutive shocks** **(*n* = 900)**	**True-refractory VF/pVT** **(*n* = 93)**	**Recurrent VF/pVT** **(*n* = 807)**	***P*-value**
Age, median (IQR)	64 (53–75)	68 (55–78)	63 (53–74)	0.079
**Age, *n* (%)**				
16–30	20 (2.2)	2 (2.2)	18 (2.2)	0.960
31–45	90 (10.0)	8 (8.6)	82 (10.2)	0.635
46–60	264 (29.3)	23 (24.7)	241 (29.9)	0.303
61–75	321 (35.7)	31 (33.3)	290 (35.9)	0.620
>75	205 (22.8)	29 (31.2)	176 (21.8)	0.041
Male sex, *n* (%)	734 (81.6)	78 (83.9)	656 (81.3)	0.543
Presumed cardiac aetiology, *n* (%)	874 (97.1)	89 (95.7)	785 (97.3)	0.391
Bystander CPR, *n* (%)*	656 (84.5)	68 (81.9)	588 (84.9)	0.487
**Witness status, *n* (%)**				
EMS	119 (13.3)	9 (9.8)	110 (13.7)	0.295
Bystander	592 (66.2)	61 (66.3)	531 (66.1)	0.973
**Arrest location, *n* (%)**				
Private residence	619 (68.8)	65 (69.9)	554 (68.7)	0.806
Public location	174 (19.3)	10 (10.8)	164 (20.3)	0.027
Aged care^a^	14 (1.6)	2 (2.2)	12 (1.5)	0.624
Other	93 (10.3)	16 (17.2)	77 (9.5)	0.022
Metropolitan region, *n* (%)	616 (68.5)	62 (66.7)	554 (68.7)	0.684
**Time variables, median (IQR)**				
EMS response time	8.2 (6.4–11.0)	8.3 (6.4–10.9)	8.2 (6.4–11.0)	0.823
Resuscitation duration	32 (17–45)	36 (21–46)	32 (16–45)	0.037
**Scene outcomes, *n* (%)**				
Died at scene or transit	378 (42.0)	49 (52.7)	329 (40.8)	0.027
Transported with CPR	62 (6.9)	8 (8.6)	54 (6.7)	0.491
Transported with ROSC	460 (51.1)	36 (38.7)	424 (52.5)	0.012
**Survival outcomes, *n* (%)**				
Pre-hospital ROSC	564 (62.7)	45 (48.4)	519 (64.3)	0.003
Event survival	463 (51.4)	37 (39.8)	426 (52.8)	0.018
Discharged alive	270 (30.3)	13 (14.0)	257 (32.2)	<0.001

CPR, cardiopulmonary resuscitation; IQR, inter-quartile range; EMS, emergency medical services, ROSC, return of spontaneous circulation.

* Exclude EMS-witnessed cases.

a Refers to residential aged care facilities.

### Adjusted impact on outcomes

The impact of true refractory VF/pVT on outcomes is shown in Table 3 (and Tables S2–3 in the supplementary Appendix). After one shock, compared to patients with recurrent VF/pVT, true-refractory VF/pVT was independently associated with lower odds of survival to hospital discharge (adjusted OR 0.53, 95% CI: 0.39–0.73, *p* < 0.001) and event survival (adjusted OR 0.70, 95% CI: 0.53–0.92, *p* = 0.010), but not prehospital ROSC (adjusted OR 0.80, 95% CI: 0.62–1.02, *p* = 0.073). After three consecutive shocks, compared to patients with recurrent VF/pVT, true-refractory VF/pVT was also associated with lower odds of survival to hospital discharge (adjusted OR 0.45, 95% CI: 0.22–0.92; *p* = 0.029) and pre-hospital ROSC (OR 0.58, 95% CI: 0.36–0.95; *p* = 0.029), but not event survival (OR 0.70, 95% CI: 0.40–1.22; *p* = 0.207).

**Table 3:** Unadjusted and adjusted impact of true-refractory VF/pVT (compared with recurrent VF/pVT) on patient outcome.

	**After 1-shock**			**After 3-shock consecutive shocks**		
	**OR**	**95% CI**	***P* value**	**OR**	**95% CI**	***P* value**
**Unadjusted**						
Survival to hospital discharge	**0.29**	**0.23**–**0.36**	**<0.001**	**0.34**	**0.19**–**0.63**	**0.001**
Event survival	**0.39**	**0.32**–**0.48**	**<0.001**	**0.59**	**0.38**–**0.92**	**0.018**
ROSC	**0.48**	**0.39**–**0.59**	**<0.001**	**0.52**	**0.34**–**0.80**	**0.003**
Adjusted*						
Survival to hospital discharge	**0.53**	**0.39**–**0.73**	**<0.001**	**0.45**	**0.22**–**0.92**	**0.029**
Event survival	**0.70**	**0.53**–**0.92**	**0.010**	0.70	0.40–1.22	0.207
ROSC	0.80	0.62–1.02	0.073	**0.58**	**0.36**–**0.95**	**0.029**

VF, ventricular fibrillation; pVT, pulseless ventricular tachycardia, OR, odd ratio; CI, confidence interval; ROSC, return of spontaneous circulation.

* Models adjusted for: age, male sex, arrest aetiology, bystander CPR, public location, metropolitan region, EMS response time and resuscitation duration.

### Factors associated with true-refractory VF/pVT

Table 4 shows the factors associated with true-refractory VF/pVT after one and three consecutive shocks. After one shock, increasing age, particularly those aged 46–60 years (OR 2.55, 95% CI: 1.08–5.91; *p* = 0.033), 61–75 years (OR 2.71, 95% CI: 1.16–6.35; *p* = 0.022), and >75 years (OR 2.41, 95% CI: 1.02–5.67; *p* = 0.045) male sex (OR 1.30, 95% CI: 1.00–1.69; *p* = 0.049) and bystander CPR (OR 1.76, 95% CI: 1.40–2.22; *p* < 0.001) were significantly associated with higher odds of true-refractory VF/pVT.

**Table 4:** Risk-adjusted odds of true-refractory VF/pVT (compared with recurrent VF/pVT) following one and three consecutive shocks.

	**After 1 shock**			**After 3 consecutive shocks**		
	**OR**	**95% CI**	***p*-value**	**OR**	**95% CI**	***p*-value**
**Age**						
16–30	Reference			Reference		
31–45	1.74	0.71–4.29	0.226	0.86	0.16–4.56	0.863
46–60	**2.52**	**1.08–5.91**	**0.033**	0.87	0.18–4.19	0.862
61–75	**2.71**	**1.16–6.35**	**0.022**	0.95	0.20–4.55	0.951
>75	**2.41**	**1.02–5.67**	**0.045**	1.44	0.30**–**6.87	0.645
**Sex**						
Female	Reference			Reference		
Male	**1.30**	**1.00**–**1.69**	**0.049**	1.37	0.76–2.47	0.296
Presumed cardiac aetiology	**0.57**	**0.34**–**0.97**	**0.037**	0.64	0.20–1.99	0.436
Bystander CPR	**1.76**	**1.40–2.22**	**<0.001**	1.19	0.71–1.98	0.510
**Arrest location**						
Other locations	Reference			Reference		
Public location	**0.71**	**0.54**–**0.94**	**0.017**	**0.47**	**0.23**–**0.93**	**0.030**
Metropolitan region	0.94	0.76–1.17	0.579	0.92	0.58–1.47	0.737
EMS response time	0.99	0.98–1.00	0.099	1.00	0.98–1.03	0.869

ROSC, return of spontaneous circulation; OR, odd ratio; CI, confidence interval; VF, ventricular fibrillation; pVT, pulseless ventricular tachycardia; CPR, cardiopulmonary resuscitation.

After three consecutive shocks, only arrests occurring in public locations remained significantly associated with lower odds of true-refractory VF/pVT (OR 0.47, 95% CI: 0.23–0.93; *p* = 0.030).

## Discussion

This study examined the impact of different definitions and phenotypes of refractory VF/pVT on survival outcomes using data from a large OHCA registry. In our cohort, the majority of patients were classified as recurrent VF/pVT, while only a minority met criteria for true-refractory VF/pVT. The proportion of all patients defined as true-refractory VF/pVT was 5.0% after three consecutive shocks and only 1.0% after five consecutive shocks. Despite this, true-refractory VF/pVT, whether defined after one or three consecutive shocks, was associated with significantly lower survival to hospital discharge compared with recurrent VF/pVT cases. While several patient and arrest characteristics were associated with true-refractory VF/pVT after the first shock, these relationships were not observed after three consecutive shocks. These findings may indicate that phenotypic differentiation of refractory VF/pVT has diminished clinical relevance beyond the initial defibrillation attempts.

The distribution of patients in our study, with only a small proportion meeting criteria for true-refractory VF/pVT, is consistent with previous studies. Studies that have involved the review of defibrillation recordings have shown that approximately 5–18% of patients remain in VF/pVT after three consecutive shocks without any termination.^4^, ^18^, ^19^ Similarly, a large multicentre observational study found that although a substantial proportion of patients met the definition of refractory VF/pVT, only 4.0% of all initially shockable arrests progressed to true shock-refractory VF/pVT.^20^ Other studies examining defibrillation response during resuscitation also support that persistent failure of VF/pVT termination is uncommon.^2^, ^21^, ^22^ Notably, our study is the first to describe the prevalence of true refractory VF/pVT across multiple defibrillation attempts up to the 15th shock, and it shows that this phenomenon is exceptionally rare, contributing only a small number of patients to the overall number of initially shockable arrests.

Emerging evidence suggests that distinguishing true-refractory from recurrent VF/pVT may be clinically meaningful.^4^, ^10^, ^23^ Studies have shown that recurrent VF/pVT, in whom VF/pVT terminates between shocks, have better outcomes than those with sustained VF/pVT (i.e., true-refractory VF/pVT).^19^, ^20^, ^23^ In the DOSE-VF trial, a double sequential defibrillation strategy targeting refractory VF/pVT was linked to improved survival, with secondary analyses suggesting that treatment effects varied by VF/pVT phenotype and were more pronounced in true-refractory VF/pVT cases.^7^, ^18^ Although these studies have defined true-refractory VF/pVT after three consecutive shocks, our analysis demonstrates that true-refractory VF/pVT is associated with poorer outcomes regardless of the time point at which it is defined. Taken together with our findings, these results indicate that early distinction between true-refractory and recurrent VF/pVT may be relevant for identifying patients who are likely to experience prolonged resuscitation trajectories and may benefit from targeted interventions.

The clinical relevance of distinguishing refractory VF/pVT phenotypes appears to be time-dependent, as true-refractory VF/pVT is uncommon and largely confined to the early phase of resuscitation. Early true-refractory VF/pVT may reflect genuine defibrillation failure rather than differences in baseline patient or arrest characteristics, shifting the attention toward factors related to shock delivery and success. One potential contributor is transthoracic impedance (TTI), which reflects chest wall resistance and influences the current delivered to the myocardium during defibrillation.^24^ Higher TTI has been associated with reduced shock effectiveness and the need for repeated shocks in other arrhythmias, such as atrial fibrillation, even with the use of impedance-compensation technology in contemporary defibrillators.^25^ Understanding how TTI influences shock success in patients with refractory VF/pVT and whether it can predict VF/pVT phenotype early in the resuscitation may facilitate consideration of interventions aimed at enhancing shock success.^26^, ^27^.

Despite the potential prognostic value of distinguishing true-refractory from recurrent VF/pVT, translating this phenotypic differentiation into real-time clinical practice remains challenging.^23^ Current resuscitation guidelines emphasise immediate resumption of chest compression after defibrillation to minimise interruptions in CPR, thereby limiting opportunities for post-shock rhythm assessment without prolonging hands-off time.^12^ Consequently, phenotypic classification of refractory VF/pVT has largely dependent on retrospective review of defibrillator recordings in observational studies and secondary analyses of clinical trials.^4^, ^10^, ^18^, ^19^, ^20^, ^23^ Emerging CPR-filtered ECG and real-time shock-feedback technologies may enable rhythm assessment during ongoing compressions, but limited validation and uptake currently constrain their clinical use.^28^ Our study suggests that if real-world differentiation is necessary, this practice could be limited to the examination of the first shock, as this would correctly identify patients who are likely to experience prolonged resuscitation attempts. Future research should focus on developing defibrillator technologies that allow reliable early differentiation of VF/pVT without interrupting chest compression.

### Limitations

This study has several limitations. First, the retrospective nature of the analysis limits our ability to infer causality and may introduce residual confounding. Second, we excluded cases where the first defibrillation was delivered by bystanders or first responders, potentially omitting early successful resuscitations and introducing selection bias. Third, our analysis did not account for treatment variables such as the use of medications or airway interventions, nor did it include pre-existing comorbidities, which may have influenced outcomes. Future studies should explore how these factors interact with VF/pVT subtypes and definitions to guide more personalised resuscitation strategies. Finally, our analysis of defibrillator recordings relied on a single senior clinician applying standardised definitions to all cases. While this approach may have introduced bias, the proportion of true-refractory VF/pVT in our cohort aligns closely with earlier studies.^19^

## Conclusion

In this population-based analysis, true-refractory VF/pVT represented a small minority of cases and was consistently associated with worse survival outcomes compared to recurrent VF/pVT when assessed after one or three consecutive defibrillation attempts. However, true-refractory VF/pVT was exceedingly rare beyond approximately five shocks, representing only 1% of all initial shockable rhythms, indicating that the distinction between these phenotypes is primarily relevant in the early phases of resuscitation and has limited relevance across the full resuscitation sequence.

## Sources of funding

ZN is supported by fellowships from the National Heart Foundation of Australia (#105690) and the National Health and Medical Research Council (#2034615).

## AI declaration for publication

During the preparation of this work, the authors used ChatGPT to improve spelling, grammar, and clarity. The authors reviewed and edited the text and take full responsibility for the final content.

## CRediT authorship contribution statement

**Abdulrahman Alhenaki:** Writing – review & editing, Writing – original draft, Methodology, Formal analysis, Conceptualization. **Zainab Alqudah:** Writing – review & editing, Supervision, Conceptualization. **Timothy Kennett:** Methodology, Data curation. **Ashanti Dantanarayana:** Methodology, Data curation. **Brett Williams:** Writing – review & editing, Supervision. **Ziad Nehme:** Writing – review & editing, Supervision, Methodology, Conceptualization.

## Declaration of competing interest

The authors declare the following financial interests/personal relationships which may be considered as potential competing interests: Given his role as a member of the editorial board of Resuscitation Plus, Ziad Nehme had no involvement in the peer review of this article and had no access to information regarding its peer review. Full responsibility for the editorial process for this article was delegated to another journal editor. If there are other authors, they declare that they have no known competing financial interests or personal relationships that could have appeared to influence the work reported in this paper.

## References

[1] Berdowski J. (2010). Global incidences of out-of-hospital cardiac arrest and survival rates: systematic review of 67 prospective studies. Resuscitation.

[2] Spies D.M. (2020). Time to change the times? Time of recurrence of ventricular fibrillation during OHCA. Resuscitation.

[3] Alhenaki A. (2024). Temporal trends in the incidence and outcomes of shock-refractory ventricular fibrillation out-of-hospital cardiac arrest. Resuscitation plus.

[4] Verkaik B.J. (2025). Defibrillation and refractory ventricular fibrillation. Eur Heart J.

[5] Kudenchuk P.J. (2016). Amiodarone, lidocaine, or placebo in out-of-hospital cardiac arrest. N Engl J Med.

[6] Suverein M.M. (2023). Early extracorporeal CPR for refractory out-of-hospital cardiac arrest. N Engl J Med.

[7] Cheskes S. (2022). Defibrillation strategies for refractory ventricular fibrillation. N Engl J Med.

[8] Yannopoulos D. (2020). Advanced reperfusion strategies for patients with out-of-hospital cardiac arrest and refractory ventricular fibrillation (ARREST): a phase 2, single centre, open-label, randomised controlled trial. Lancet.

[9] Yannopoulos D. (2016). Minnesota resuscitation consortium's advanced perfusion and reperfusion cardiac life support strategy for out-of-hospital refractory ventricular fibrillation. J Am Heart Assoc.

[10] Shanmugasundaram M. (2012). Analysis of amplitude spectral area and slope to predict defibrillation in out of hospital cardiac arrest due to ventricular fibrillation (VF) according to VF type: recurrent versus shock-resistant. Resuscitation.

[11] Holmén J. (2017). Survival in ventricular fibrillation with emphasis on the number of defibrillations in relation to other factors at resuscitation. Resuscitation.

[12] Soar J., Guidelines E.R.C. (2025). Adult advanced life support. Resuscitation.

[13] Nehme Z. (2019). Reply to: Importance of the distinction between recurrent and shock-resistant ventricular fibrillation: call for a uniform definition of refractory VF. Resuscitation.

[14] Nehme E. (2024). Out-of-hospital cardiac arrests in Victoria, 2003–2022: retrospective analysis of Victorian Ambulance Cardiac Arrest Registry data. Med J Aust.

[15] Nair R, Andrew E, Sathish-Kumar K. Cardiac arrest registry 2020–2021 annual report; 2020–21.

[16] Matsuyama T. (2022). Cardiopulmonary resuscitation duration and favorable neurological outcome after out-of-hospital cardiac arrest: a nationwide multicenter observational study in Japan (the JAAM-OHCA registry). Crit Care.

[17] Alhenaki A. (2025). Impact of resuscitation duration on 12-month functional recovery following out-of-hospital cardiac arrest with initially shockable rhythms. Resuscitation.

[18] Cheskes S. (2024). The impact of alternate defibrillation strategies on shock-refractory and recurrent ventricular fibrillation: a secondary analysis of the DOSE VF cluster randomized controlled trial. Resuscitation.

[19] Verkaik B.J. (2023). Abstract 419: incidence of true refractory ventricular fibrillation in patients meeting a pragmatic definition of refractory ventricular fibrillation. Circulation.

[20] Magliocca A. (2025). Amplitude spectrum area to predict true shock-refractory ventricular fibrillation during basic life support-treated out-of-hospital cardiac arrest. Resuscitation.

[21] Pandit S.V., Lampe J.W., Silver A.E. (2024). Recurrence of ventricular fibrillation in out-of-hospital cardiac arrest: clinical evidence and underlying ionic mechanisms. J Physiol.

[22] Koster R.W., Walker R.G., Chapman F.W. (2008). Recurrent ventricular fibrillation during advanced life support care of patients with prehospital cardiac arrest. Resuscitation.

[23] Nas J. (2019). Importance of the distinction between recurrent and shock-resistant ventricular fibrillation: call for a uniform definition of refractory VF. Resuscitation.

[24] Heyer Y., Baumgartner D., Baumgartner C. (2022). A systematic review of the transthoracic impedance during cardiac defibrillation. Sensors (Basel).

[25] Sadek M.M. (2018). Association between transthoracic impedance and electrical cardioversion success with biphasic defibrillators: an analysis of 1055 shocks for atrial fibrillation and flutter. Clin Cardiol.

[26] Voskoboinik A. (2022). First time use of manual pressure augmentation for ventricular fibrillation arrest in the community. Resuscitation.

[27] Zhang B. (2014). Anterior-posterior versus anterior-lateral electrode position for external electrical cardioversion of atrial fibrillation: a meta-analysis of randomized controlled trials. Arch Cardiovasc Dis.

[28] Gong Y., Chen B., Li Y. (2013). A Review of the performance of artifact filtering algorithms for cardiopulmonary resuscitation. J Healthc Eng.

