# Supplementary material for: A comparison of the epidemiology and outcomes of true refractory and recurrent ventricular fibrillation out-of-hospital cardiac arrest: a retrospective study
Source: Resusc Plus. 2026 Mar 27;29:101302. doi: 10.1016/j.resplu.2026.101302 (PMC13087690; doi:10.1016/j.resplu.2026.101302)
Supplement: Supplementary Tables [file mmc1.docx]

**Table S1: Rhythm definitions applied in the study.**

| **Rhythm** | **Definition used** |
| --- | --- |
| **Ventricular Fibrillation** | Chaotic variations in baseline ≥0.1mV |
| **Ventricular Tachycardia** | A regular broad complex (>0.12 sec) tachycardia of >100/min. Torsades de pointes and polymorphic VT are acceptable variants. |
| **Pulseless Electrical Activity or organised rhythm** | An organised or semi-organised rhythm with QRS complexes which would normally be expected to produce circulation. |
| **Asystole** | Variations in baseline of <0.1mV |
| **Unknown** | Rhythm cannot be determined due to artefact |

**Table S2 Adjusted impact of true-refractory VF/pVT (compared to recurrent VF/pVT) on patient outcomes after one shock**

|  | Survival to  hospital discharge | | | Event survival | | | Pre-hospital ROSC | | |
| --- | --- | --- | --- | --- | --- | --- | --- | --- | --- |
|  | **OR** | **95% CI** | **P value** | **OR** | **95% CI** | **P value** | **OR** | **95% CI** | **P value** |
| True-refractory VF/pVT | **0.53** | **0.39-0.73** | **<0.001** | **0.70** | **0.53-0.92** | **0.010** | 0.80 | 0.62-1.02 | 0.073 |
| Age |  |  |  |  |  |  |  |  |  |
| 16 – 30 | Reference | | | Reference | | | Reference | | |
| 31 – 45 | **0.27** | **0.09-0.82** | **0.021** | 0.40 | 0.14-1.17 | 0.094 | 0.69 | 0.26-1.80 | 0.446 |
| 46 – 60 | **0.25** | **0.09-0.71** | **0.009** | 0.39 | 0.14-1.09 | 0.072 | 0.68 | 0.27-1.70 | 0.412 |
| 61 – 75 | **0.13** | **0.04-0.37** | **<0.001** | **0.25** | **0.09-0.71** | **0.009** | 0.45 | 0.18-1.11 | 0.082 |
| >75 | **0.03** | **0.01-0.10** | **<0.001** | **0.13** | **0.05-0.37** | **<0.001** | **0.28** | **0.11-0.70** | **0.007** |
| Gender |  |  |  |  |  |  |  |  |  |
| Female | Reference | | | Reference | | | Reference | | |
| Male | 0.95 | 0.69-1.32 | 0.772 | 0.95 | 0.69-1.32 | 0.777 | 0.94 | 0.69-1.27 | 0.679 |
| Presumed cardiac aetiology | **6.82** | **3.06-15.21** | **<0.001** | **2.02** | **1.05-3.87** | **0.035** | **1.85** | **1.01-3.37** | **0.046** |
| Bystander CPR | **0.63** | **0.47-0.85** | **0.003** | 1.03 | 0.76-1.39 | 0.869 | 0.80 | 0.61-1.06 | 0.121 |
| Arrest location |  |  |  |  |  |  |  |  |  |
| Other locations | Reference | | | Reference | | | Reference | | |
| Public location | **2.32** | **1.62-3.31** | **<0.001** | 1.11 | 0.79-1.57 | 0.537 | 1.19 | 0.87-1.62 | 0.288 |
| Metropolitan region | 1.09 | 0.82-1.45 | 0.559 | 0.96 | 0.73-1.27 | 0.774 | 1.03 | 0.80-1.32 | 0.818 |
| EMS response time | 1.00 | 0.99-1.01 | 0.989 | 1.00 | 0.99-1.01 | 0.758 | 1.01 | 0.99-1.02 | 0.449 |
| CPR duration | **0.90** | **0.89-0.90** | **<0.001** | **0.90** | **0.89-0.91** | **<0.001** | **0.93** | **0.92-0.94** | **<0.001** |
| ROSC, return of spontaneous circulation; OR, odd ratio; CI, confidence interval; VF, ventricular fibrillation; pVT, pulseless ventricular tachycardia; CPR, cardiopulmonary resuscitation. | | | | | | | | | |

**Table S3 Adjusted impact of true-refractory VF/pVT (compared to recurrent VF/pVT) on patient outcomes after three consecutive shocks**

|  | Survival to  hospital discharge | | | Event survival | | | Pre-hospital ROSC | | |
| --- | --- | --- | --- | --- | --- | --- | --- | --- | --- |
|  | **OR** | **95% CI** | **P value** | **OR** | **95% CI** | **P value** | **OR** | **95% CI** | **P value** |
| True-refractory VF/pVT | **0.45** | **0.22-0.92** | **0.029** | 0.70 | 0.40-1.22 | 0.207 | **0.58** | **0.36-0.95** | **0.029** |
| Age |  |  |  |  |  |  |  |  |  |
| 16 – 30 | Reference | | | Reference | | | Reference | | |
| 31 – 45 | **0.22** | **0.05-0.94** | **0.041** | 0.58 | 0.15-2.23 | 0.426 | 0.99 | 0.29-3.39 | 0.993 |
| 46 – 60 | **0.19** | **0.05-0.76** | **0.019** | 0.39 | 0.11-1.43 | 0.156 | 0.77 | 0.24-2.45 | 0.655 |
| 61 – 75 | **0.13** | **0.03-0.53** | **0.004** | 0.34 | 0.09-1.23 | 0.100 | 0.56 | 0.17-1.78 | 0.324 |
| >75 | **0.03** | **0.01-0.14** | **<0.001** | **0.19** | **0.05-0.71** | **0.013** | 0.45 | 0.14-1.47 | 0.187 |
| Gender |  |  |  |  |  |  |  |  |  |
| Female | Reference | | | Reference | | | Reference | | |
| Male | 0.85 | 0.52-1.39 | 0.521 | 1.08 | 0.69-1.71 | 0.734 | 0.98 | 0.65-1.47 | 0.925 |
| Presumed cardiac aetiology | **5.01** | **1.33-18.88** | **0.017** | 0.88 | 0.31-2.47 | 0.803 | 0.85 | 0.32-2.23 | 0.743 |
| Bystander CPR | 1.08 | 0.69-1.69 | 0.728 | 1.05 | 0.69-1.59 | 0.831 | 0.87 | 0.60-1.27 | 0.479 |
| Arrest location |  |  |  |  |  |  |  |  |  |
| Other locations | Reference | | | Reference | | | Reference | | |
| Public location | **1.83** | **1.15-2.92** | **0.011** | 0.90 | 0.57-1.43 | 0.664 | 1.04 | 0.69-1.56 | 0.844 |
| Metropolitan region | 1.18 | 0.79-1.77 | 0.429 | 0.82 | 0.56-1.19 | 0.297 | 0.92 | 0.66-1.29 | 0.634 |
| EMS response time | 1.01 | 0.98-1.03 | 0.613 | **0.98** | **0.96-1.00** | **0.037** | 1.00 | 0.98-1.02 | 0.789 |
| CPR duration | **0.90** | **0.89-0.91** | **<0.001** | **0.90** | **0.88-0.91** | **<0.001** | **0.94** | **0.93-0.95** | **<0.001** |
| ROSC, return of spontaneous circulation; OR, odd ratio; CI, confidence interval; VF, ventricular fibrillation; pVT, pulseless ventricular tachycardia; CPR, cardiopulmonary resuscitation. | | | | | | | | | |
